# Isolation, Molecular Characterization, and Mapping of Four Rose *MLO* Orthologs

**DOI:** 10.3389/fpls.2012.00244

**Published:** 2012-11-02

**Authors:** Helgard Kaufmann, Xianqin Qiu, Juliane Wehmeyer, Thomas Debener

**Affiliations:** ^1^Department of Molecular Breeding, Institute for Plant Genetics, Leibniz University of HannoverHannover, Germany; ^2^Yunnan Flower Breeding Laboratory, Flower Research Institute, Yunnan Academy of Agricultural SciencesKunming, China

**Keywords:** powdery mildew, mildew resistance locus o, *Podosphaera pannosa*, rosaceae, tetraploid

## Abstract

Powdery mildew is a major disease of economic importance in cut and pot roses. As an alternative to conventional resistance breeding strategies utilizing single-dominant genes or QTLs, mildew resistance locus o (*MLO*)-based resistance might offer some advantages. In dicots such as *Arabidopsis*, pea, and tomato, loss-of-function mutations in *MLO* genes confer high levels of broad-spectrum resistance. Here, we report the isolation and characterization of four *MLO* homologs from a large rose EST collection isolated from leaves. These genes are phylogenetically closely related to other dicot *MLO* genes that are involved in plant powdery mildew interactions. Therefore, they are candidates for *MLO* genes involved in rose powdery mildew interactions. Two of the four isolated genes contain all of the sequence signatures considered to be diagnostic for *MLO* genes. We mapped all four genes to three linkage groups and conducted the first analysis of alternative alleles. This information is discussed in regards to a reverse genetics approach aimed at the selection of rose plants that are homozygous for loss-of-function in one or more *MLO* genes.

## Introduction

Powdery mildew is one of the most serious rose diseases. Disease control is economically important for cut and pot roses, which are almost exclusively produced in greenhouses throughout temperate regions or at higher elevations in the tropics. The application of fungicides to combat powdery mildew is expensive and, in most parts of the world, increasingly limited by legal restrictions. These circumstances leave genetic strategies as the only environmentally friendly alternative for controlling disease.

Powdery mildew of roses is caused by the obligate biotrophic ascomycete *Podosphaera pannosa* (formerly *Sphaerotheca pannosa*, Linde and Shishkoff, 2003). *P. pannosa* belongs to the family *Erysiphaceae*, which consists of other important powdery mildews such as *Blumeria graminis* of cereals. The distribution of conidia occurs almost exclusively by wind over long distances. Under favorable conditions conidia germinate 2–6 h after deposition on the plant. Germ tubes develop an appressorium which forms a penetration peg to breach the cell wall. After successful penetration a haustorium is formed 1 h after infection. After successful establishment of haustoria additional mycelium is formed that spreads across the leaf surface forming new appressoria. Approximately 3–5 days after infection new conidiophores are formed completing the vegetative life cycle. In addition to the vegetative life cycle *P. pannosa* also forms sexual spores in so-called “ascomata” under less favorable conditions. Heavily infected leaves display distortions and often die but even mildly infected leaves reduce the esthetic value of a rose plants making cut and pot roses non-marketable.

Studies of the interactions between rose plants and powdery mildew revealed that several pathogenic races of the fungus exist, and both qualitative and quantitative resistance loci are present in the host (Linde and Debener, 2003; Leus et al., 2006). Linde and Debener (2003) distinguished eight pathogenic races among eight isolates tested whereas Leus et al. (2006) identified seven pathogenic races. To date, three single major resistance genes have been identified in diploid roses (Xu et al., 2005; Debener and Linde, 2009). In a number of studies, several major QTL regions for resistance were localized on linkage maps for both diploid and tetraploid populations (Dugo et al., 2005; Linde et al., 2006; Hosseini Moghaddam et al., 2012). The large number of pathogenic races identified within a relatively small sample of isolates indicates that a large genetic diversity exists in this pathogen compared to other well-characterized powdery mildews (Muller et al., 1996). Monogenic resistance factors may lead to so-called boom and bust cycles (Thompson and Burdon, 1992), which render resistance ineffective within a short time period. The use of quantitative resistance genes as alternatives to monogenic resistance genes is hampered by the tetraploid nature of most cultivated roses. Tetraploidy leads to an extremely complex inheritance of resistance QTLs. A rarely exploited alternative to combat powdery mildew diseases is mildew resistance locus o (*MLO*)-based resistance in plants (Buschges et al., 1997; Consonni et al., 2006; Humphry et al., 2006; Bai et al., 2008; Pavan et al., 2011).

Mildew resistance locus o-based resistance was characterized several decades ago in barley as a recessive inheritance that is effective against all known races of barley powdery mildew (Jorgensen, 1992). The MLO protein belongs to a plant-specific family of membrane proteins that contain seven transmembrane helices and a C-terminal calmodulin-binding domain (Devoto et al., 1999; Kim et al., 2002). Although the exact biological functions of MLO proteins remain elusive, some family members may regulate penetration resistance via control of vesicle fusion events (Collins et al., 2003; Bhat et al., 2005). In natural or induced barley mutants with *MLO* loss-of-function, powdery mildew penetration rates are greatly reduced. *MLO* orthologs have been characterized in dicots such as *Arabidopsis*, *Pisum*, and *Solanum* (Consonni et al., 2006; Bai et al., 2008; Humphry et al., 2011; Pavan et al., 2011). In pea and tomato, recessive mutants of single *MLO* genes confer full broad-spectrum resistance to powdery mildew. The loss-of-function of three *MLO* orthologs (*MLO* 2, 6, and 12) is needed for resistance in *Arabidopsis thaliana*.

The observation that mutant MLO proteins confer powdery mildew resistance in monocot and dicot species indicates that the function of this protein in the plant powdery mildew interaction is highly conserved. *MLO* genes provide a general route to achieving highly effective race-independent resistance in angiosperms.

Reverse genetics is a possible strategy for developing *MLO*-based resistance in dicots for which no mutants are currently known. In the event that *MLO* orthologs have been identified, a large screen for mutant alleles in either mutagenized or natural populations can be undertaken to search for likely loss-of-function variants. Comparable screens have been conducted in a number of species by TILLING (reviewed in Kurowska et al., 2011) or next-generation sequencing (Marroni et al., 2011; Tsai et al., 2011). Therefore, laborious infection experiments can be restricted to candidate genotypes that contain loss-of-function mutations.

The aims of this study were to identify the rose orthologs of MLO protein family members that are involved in the interaction with powdery mildew pathogens and to analyze the complexity of these orthologs within the rose genome. In future studies, this information can be used to perform large-scale mutation screens for loss-of-function variants.

## Materials and Methods

### Plant materials

Three diploid *R. multiflora* hybrids, 88/124-46, 82/78-1, and 97/7-185 (Linde et al., 2004), and the tetraploid *R. hybrida* cv. “Pariser Charme” were used in this study to isolate cDNA and genomic MLO sequences. The diploid rose mapping population 97/7 (95/13-39 × 82/78-1) of 270 plants has been described previously (Linde et al., 2006). Plants were cultivated either in the greenhouse under semi-controlled conditions or in the field.

### EST library, isotigs, and BLAST analysis

ESTs with similarities to *MLO* genes were obtained from a collection of ESTs generated from genotype 88/124-46. In brief, the following tissues and treatments were used: untreated leaves, leaves inoculated with compatible and incompatible isolates of black spot (*Diplocarpon rosae*), leaves inoculated with compatible isolates of powdery mildew (*P. pannosa*) and downy mildew (*Peronospora sparsa*), leaves wounded by scalpels and leaves treated by heat shock at 40°C for 1 h. Two normalized cDNA libraries were generated by Vertis Biotechnologie AG (Weihenstephan, Germany). One library (library A) consisted of cDNA generated from RNA from untreated leaves. Another library (library B) was made from cDNA generated from equal amounts of RNA from each of the stress treatments. Normalized cDNA was ligated to the 454 adaptors and amplified by nine PCR cycles. A size fraction of 500–700 bp was sequenced by Roche using the 454 Titanium method. Raw reads were filtered and trimmed. Totals of 1,173,352 reads from library A and 1,148,031 reads from library B were assembled together using the Newbler assembler (version 2.3) run in “cDNA mode” and “overlapMInMatchIdentity = 95%.” For all other parameters, standard settings were used.

The resulting assembly consists of 52,223 contigs, which could be further grouped into 44,343 isotigs (including splice variants). All searches were made at the level of the assembled isotigs.

### Cloning of *MLO* sequences

#### Isolation of DNA and RNA

DNA and RNA were extracted from young rose leaves as described by Terefe-Ayana et al. (2011).

#### PCR primers

Primers were designed using Primer3^1^. All primers used in this study are listed in Table A1 in Appendix.

#### RT-PCR and rapid amplification of cDNA ends

First-strand cDNAs were synthesized by oligo-dT priming with a high-capacity cDNA reverse transcription kit (Applied Biosystems, Life Technologies). For 3′RACE reactions, a tailed oligo-dT adapter primer (AP, Invitrogen, Life Technologies) was used. The 3′RACE PCR fragments were amplified in nested PCR reactions with an abridged universal amplification primer (AUAP, Invitrogen, Life Technologies) and two gene-specific primers. The 3′RACE fragments and full length *MLO* cDNAs were amplified with PrimeSTAR HS DNA polymerase (TAKARA Bio Europe). The 5′ RACE reactions were performed with the FirstChoice RLM-RACE Kit (Ambion, Life Technologies) according to the manufacturer’s instructions using Phusion High-Fidelity DNA polymerase (Finnzymes, Biozym Scientific GmbH, Oldendorf, Germany) for PCR.

#### Genomic PCR

Reactions were performed following the supplier’s instructions with Phusion High-Fidelity DNA polymerase (Finnzymes, Biozym Scientific GmbH, Oldendorf, Germany) for *MLO* fragments up to 6 kb and with LongAmp Taq DNA polymerase (New England Biolabs) for fragments above 6 kb.

#### Cloning of PCR products

PCR fragments were separated by size on agarose gels and isolated with a Gel/PCR DNA fragment extraction kit (Avegene, DCS Hamburg, Germany). Fragments were then cloned into either pJET1.2 using the Clone Jet PCR cloning kit (Fermentas, Germany) or pGEM-T-Easy (Promega Germany) after A-tailing, according to the kit protocol.

### Bioinformatics

BioEdit 7.0.9.0 (Hall, 1999) was used for DNA sequence manipulations, assemblies, and BLAST analyses (Altschul et al., 1997) in local databases. BLASTn and BLASTx searches were also performed against the GenBank database^2^ and against the *Prunus persica* and *Fragaria vesca* genomes in the Genome Database for Rosaceae^3^.

For the phylogenetic analyses of MLO proteins, HvMLO (GenBank Accession number Z83834), AtMLO1 (Z95352), AtMLO2–AtMLO15 (AF369563–AF369576) and MLO orthologs TaMLO2 (AF361932), SlMLO1 (AAX77013), PsMLO1 (FJ463618), MtMLO1 (HQ446457), and LjMLO1 (AY967408) downloaded from GenBank were aligned with RhMLO1–RhMLO4 (JX847131–JX847134) by ClustalW using default parameters (Thompson et al., 1994)^4^.

Different amino acid substitution models were tested in Mega5 (Tamura et al., 2011) and the Jones Taylor Thornton correction model (JTT) with evolutionary rates following a discrete gamma distribution (+G) was chosen as the most likely model.

The alignment was used to generate a phylogenetic tree in Mega5 (Tamura et al., 2011) using the Maximum Likelihood method based on the JTT+G model. All positions containing gaps and missing data were eliminated. There were a total of 395 positions in the final dataset. Support for the branching was evaluated by bootstrap analysis with 500 replications. In addition to the maximum likelihood method neighbor joining and minimum evolution trees were inferred in Mega5 from 500 bootstrap replicates using the same substitution model and amino acid positions as described above.

### Marker development and mapping

Primers for four rose *MLO* genes, RhMLO1–4, were designed based on the genomic *MLO* sequences of genotype 88/124-46. PCR amplifications were performed under the conditions described in Biber et al. (2010). For RhMLO1 and RhMLO2, sequence polymorphisms of the PCR products were analyzed using the single-stranded conformation polymorphism (SSCP) method (Orita et al., 1989) as described by Yan et al. (2005). A polymorphism in the length of RhMLO3 PCR fragments was detected with a Li-COR DNA-Analyzer Gene Read 4200 (LI-COR, Inc.) using IRD700 labeled primers (Biber et al., 2010). The PCR fragments of RhMLO4 were digested with *Hsp*92II (Promega) to be analyzed as cleaved amplified polymorphic sequences (CAPS) on 2.5% agarose gels. RhMLO1, 2, and 4 were scored as uni-parental markers for the parent 95/13-39; RhMLO3 was scored as a bi-parental marker in the population 97/7. The genetic linkage map for 95/13-39 was recalculated using JoinMap4.0 (van Ooijen, 2006). Settings were as described by Spiller et al. (2011).

## Results

### Screen of the ESTs

To obtain rose orthologs of *MLO*, 44,343 isotigs from the EST collection of the genotype 88/124-46 were screened by BLASTx searches against the *Arabidopsis* *MLO* genes 2, 6, and 12. A total number of 31 isotigs with significant matches was obtained. These were used to perform BLAST searches against a local database consisting of all known *Arabidopsis* *MLO* sequences. Two isogroups with four to five isotigs each and two single isotigs had more significant matches to the *Arabidopsis* *MLO* genes 2, 6, and 12 than to other *MLO* genes. This result indicated that these rose sequences might be true orthologs of the *MLO* cluster. Based on these sequences, four *Rosa hybrida* *MLO* ortholog candidates were named RhMLO1 to RhMLO4 and were analyzed in more detail.

### Generation of full length cDNA clones

Only one of the isogroups representing the rose *MLO* sequences consisted of the full coding sequence. The coverage of the original reads differed between the sequences. Different strategies were pursued to obtain full length cDNA sequences for RhMLO1–4.

For RhMLO1, four isotigs representing the complete coding sequences of two alleles enabled us to perform RT-PCR with a primer combination binding to the 5′ and 3′untranslated region. We obtained a single distinct product of the expected size that was cloned and sequenced.

No complete coding sequence for RhMLO2 was available in the rose EST library. An orthologous gene was identified by BLASTx and BLASTn searches using individual rose ESTs against *F. vesca* coding sequences in the GDR *Rosaceae* database. These searches identified two ESTs covering the 5′ and 3′ ends of the rose homolog of the gene 09653 in *Fragaria*. Based on the sequences of these ESTs, the full length cDNA was obtained by RT-PCR.

RhMLO3 and RhMLO4 were identified and cloned by RT-PCR amplification of a core fragment, followed by 5′ and 3′ RACE to obtain full length sequence information. This procedure was necessary because an attempt to obtain a full length cDNA from the underlying set of five isotigs failed. A core fragment of 1 kb was obtained based on the sequence information from these isotigs. The fragment contained two clearly different but closely related sequences. 5′ and 3′RACE reactions were performed with primers from conserved regions of this *MLO* fragment. The reactions resulted in the sequence characterization of RhMLO3 and RhMLO4 in three overlapping fragments. The sequences obtained were verified by the amplification of the complete coding sequences in one RT-PCR fragment. Although the MLO3 and MLO4 DNA sequences share similarities of 94% compared to DNA sequence identities between 59 and 60% with MLO1 and MLO2, they represent two different genes. This finding was confirmed by the numbers of alleles isolated from several diploid rose genotypes, which exceeded two sequences for the genotypes 82/78-1 and 97/7-185. Although both genes are tightly linked, they are separated by recombinants on the rose genetic map.

In summary, we have obtained complete coding sequences from rose leaf RNA for the four *MLO* candidate genes RhMLO1–RhMLO4.

### Phylogenetic analysis of rose MLO sequences

**Figure 1:**
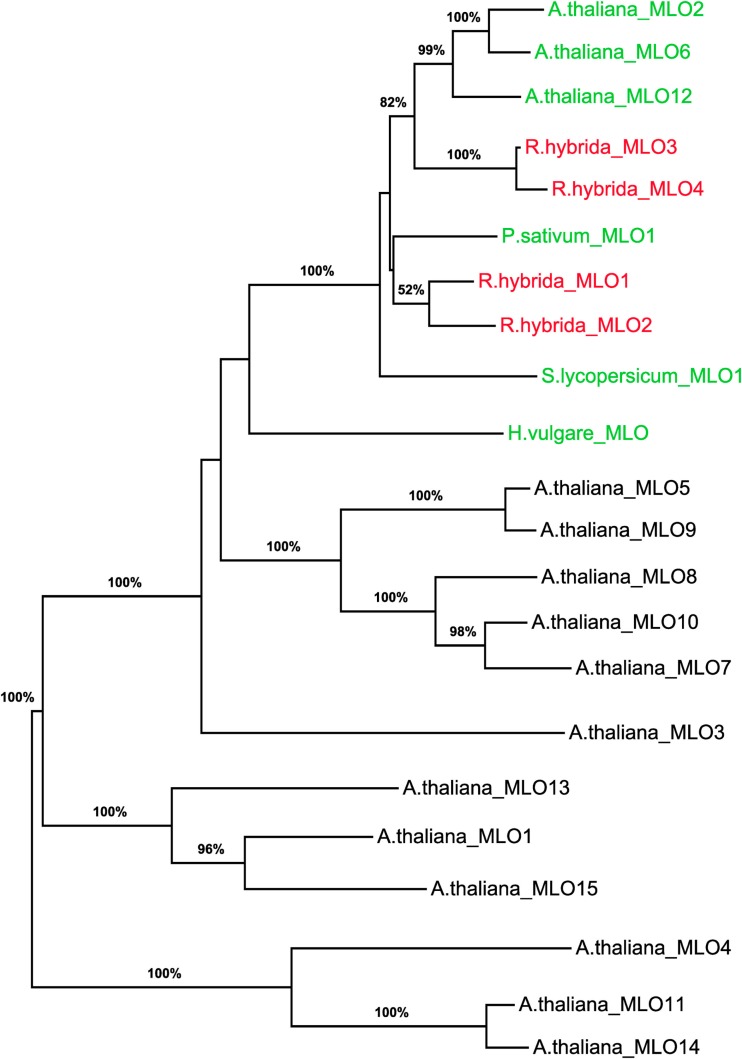
**Molecular phylogenetic analysis of the four rose *MLO* genes**. A maximum likelihood phylogenetic tree of the amino acid sequences of RhMLO1–4, the *Arabidopsis thaliana* MLO family (Devoto et al., 2003) and MLO orthologs of barley, tomato, and pea (Buschges et al., 1997; Bai et al., 2008; Humphry et al., 2011) is shown. Numbers at the branch nodes indicate bootstrap values from 500 replications. Branch length is proportional to sequence distance. The clades containing the MLO orthologs involved in plant powdery mildew interactions are indicated by colored gene names. The names of the rose MLO sequences are printed in red. GenBank Accession numbers of the MLO genes encoding the respective proteins are as follows: RhMLO1–4 (JX847131–JX847134), HvMLO (Z83834), AtMLO1 (Z95352), AtMLO2–AtMLO15 (AF369563–AF369576), PsMLO1 (FJ463618), SlMLO1 (AAX77013).

**Figure 2:**
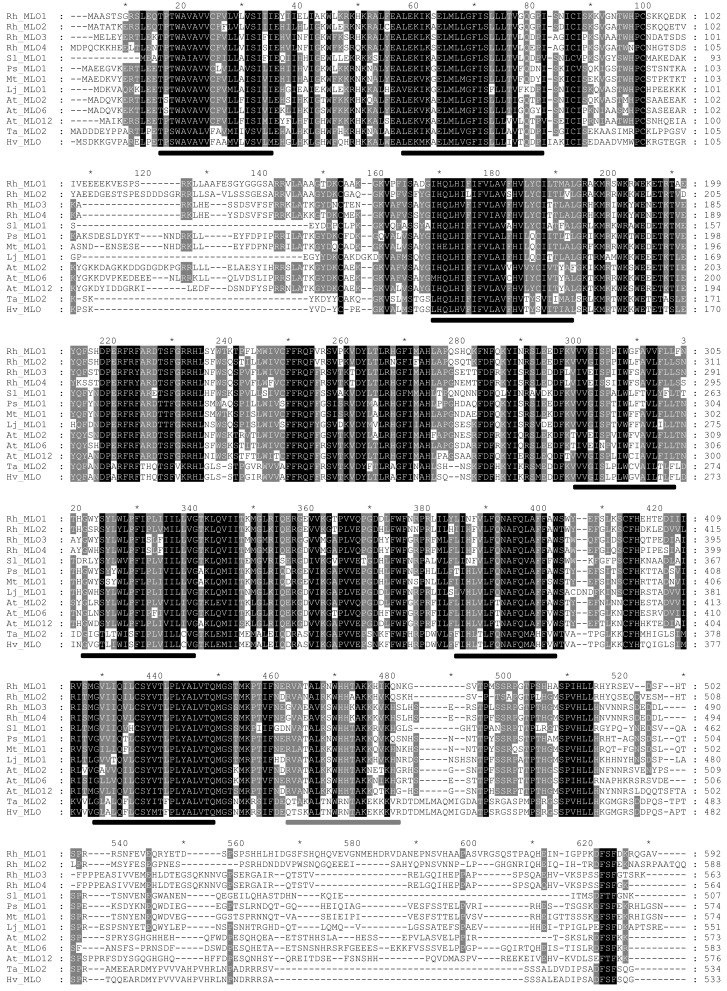
**Multiple sequence alignment of RhMLO proteins with selected MLO proteins that have been shown to be involved in susceptibility to powdery mildew in *Arabidopsis* (AtMLO2, AtMLO6, AtMLO12; Consonni et al., 2006), barley (HvMLO; Buschges et al., 1997), tomato (SlMLO1; Bai et al., 2008), pea, *Medicago* (PsMLO1, MtMLO1, LjMLO1; Humphry et al., 2011), and wheat (TaMLO2; Devoto et al., 2003)**. The alignment was generated by ClustalW using default parameters. Positions of the seven transmembrane domains (Devoto et al., 1999) are indicated by black bars below the sequences. The position of the calmodulin-binding site (Panstruga, 2005) is indicated by a gray bar. GenBank accessions of translated sequences: RhMLO1–4 (JX847131–JX847134), AtMLO2 (AF369563), AtMLO6 (AF369567), AtMLO12 (AF369573), HvMLO (Z83834), SlMLO1 (AAX77013), PsMLO1 (FJ463618), MtMLO1 (HQ446457), TaMLO2 (AF361932).

To determine whether RhMLO1, 2, 3, and 4 represent orthologs of HvMLO, SlMLO1, PsMLO1, and AtMLO2, 6, and 12 (Buschges et al., 1997; Consonni et al., 2006; Bai et al., 2008; Humphry et al., 2011; Pavan et al., 2011), we constructed a phylogenetic tree of the corresponding amino acid sequences and the AtMLO homologs AtMLO1 to AtMLO15. The four rose MLO proteins clustered with the dicot MLO proteins and are therefore genuine *MLO* candidates (Figure 1). Their position in the dendrogram is supported by high bootstrap values and is independent of the methods for phylogenetic reconstruction (data not shown). This is in accordance with the data for amino acid identities between the sequences. These are larger than 0.5 for the rose MLOs compared to the characterized dicot MLO involved in plant powdery mildew interactions and lower than 0.4 between the rose sequences and all other Arabidopsis MLO genes (Table A2 in Appendix). A more detailed sequence comparison in a ClustalW alignment of the MLO orthologs was conducted that included the recently identified *MLO* orthologous genes from *Medicago trunculata* and *Lotus japonicus* (Humphry et al., 2011) and the monocot ortholog TaMLO2 (Devoto et al., 2003). This allowed for the examination of the rose MLO proteins for domains that are conserved between all proteins responsible for mildew susceptibility. This alignment depicted only a few positions that are conserved between all orthologs except rose. A similar result was obtained for individual dicot orthologs. The deviation of the C-terminal peptide motif S-F-S-F in RhMLO3 and 4 from the consensus D/E-F-S/T-F is noticeable. Due to the small number of functionally characterized MLO proteins, we cannot exclude that these variants are also functional MLO proteins. Other domains have been shown to be essential for MLO function. The transmembrane domains and the calmodulin-binding site (Devoto et al., 1999; Elliott et al., 2002; Kim et al., 2002; Panstruga, 2005; Reinstaedler et al., 2010) are also conserved in RhMLO1–4 (Figure 2). In conclusion, all four rose genes may code for functional MLO proteins involved in plant-pathogen interactions.

### Alleles of RhMLO1–4

To analyze the sequence variability in rose *MLO* orthologs, we amplified via RT-PCR and sequenced alleles of RhMLO1–4 from two additional diploid rose genotypes and from the tetraploid rose cultivar “Pariser Charme.” We obtained six alleles for RhMLO1, two alleles for RhMLO2, four alleles for RhMLO3 and five alleles for RhMLO4. The number of alleles obtained and their sequence identities reflect the differences in the conservation of these genes. The extent of similarity between the alleles in the protein coding sequence ranged from 0.957 to 1.000, with indels (insertion/deletion) and single nucleotide polymorphism (SNPs) causing the polymorphisms (Table 1). The highest conservation was observed in RhMLO2; only two alleles were identified with sequence similarities of 99.6%. The variability is high in RhMLO4 due to two alleles with deletions in the 3′ region of the sequence. For none of the alleles of RhMLO1-3 any indication for non-sense mutations or premature stop codons was found. For RhMLO4 two alleles carry indels that cause premature stops. The resulting proteins are truncated at amino acid positions 519 and 532 respectively. However, it remains to be shown whether this has any consequences for the function of the proteins.

**Table 1:** **Alleles of RhMLO1–RhMLO4 that were isolated from four rose genotypes**.

Gene	Number of different alleles	Length in bp from ATG to STOP	Sequence identity in % (DNA sequence)	Number of indels	Number of SNPs	Number of sense-/non-sense mutations in protein
RhMLO1	6	1776	97.7–99.7	1	69	28/−
RhMLO2	2	1767	99.7	−	6	2/−
RhMLO3	4	1692	97.6–99.2	−	53	33/−
RhMLO4	5	1695	95.7–100^1^	3	49	40/2^2^

*^1^SNPs in non-translated regions*.

*^2^Premature stop: deletion of 33 amino acids/46 amino acids at the C termini*.

### Structural organization of Rh*MLO* genomic sequences

**Figure 3:**
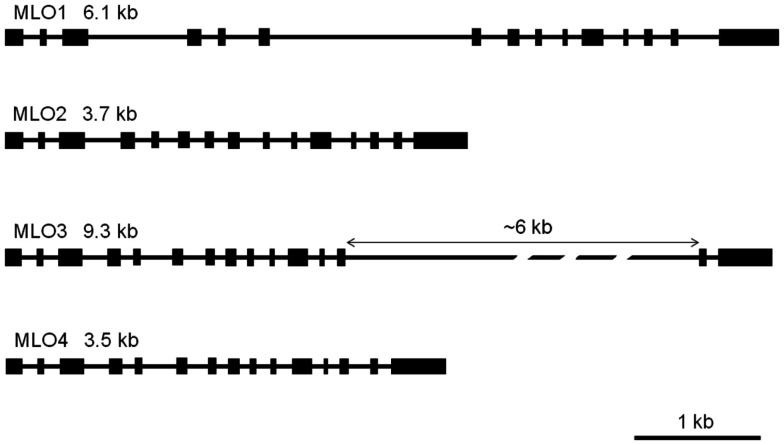
**Gene structures of the four rose *MLO* genes**. The genes are built from 15 exons, depicted as boxes, that are interrupted by introns of varying size (black bars). The proportions reflect the actual sizes of introns and exons except for the 6 kb intron in MLO3. Gene sizes are given from ATG to STOP in kb.

As a prerequisite for genome comparison, mapping, and future TILLING approaches, the genomic structures of RhMLO1–4 were investigated in the *R. multiflora* hybrid 88/124-46. All *MLO* genes analyzed thus far are composed of 12–15 exons that are interrupted at conserved positions mostly by short introns of approximately 100 nucleotides (Buschges et al., 1997; Devoto et al., 2003). The genomic sequences of RhMLO1–RhMLO4 are built from 15 exons with introns at identical positions and therefore contain the same general structure as other *MLO* genes. We did not detect any intron in the UTR regions of the rose genes. Due to differences in intron sizes, the total lengths of the rose genes range from 3.5 kb (RhMLO4) to 9.3 kb (RhMLO3). A comparison of the gene structures is shown in Figure 3. RhMLO2 and RhMLO4 show a standard gene size, with small introns comparable to *Arabidopsis* and barley *MLO*s. RhMLO1 contains two larger introns (introns 3 and 6) with lengths of 0.8 and 1.6 kb, respectively. The 6 kb intron 13 in RhMLO3 is caused by a retroposon insertion. Despite this large insertion, the intron is spliced correctly in the corresponding cDNA. A comparison of genomic *MLO* PCR products from a random sampling of rose genotypes revealed a conservation of gene size for RhMLO1, 2, and 4. Two gene sizes (with or without a retroposon) were identified for RhMLO3 (data not shown).

### Localization of RhMLO1–4 in the rose genome

**Figure 4:**
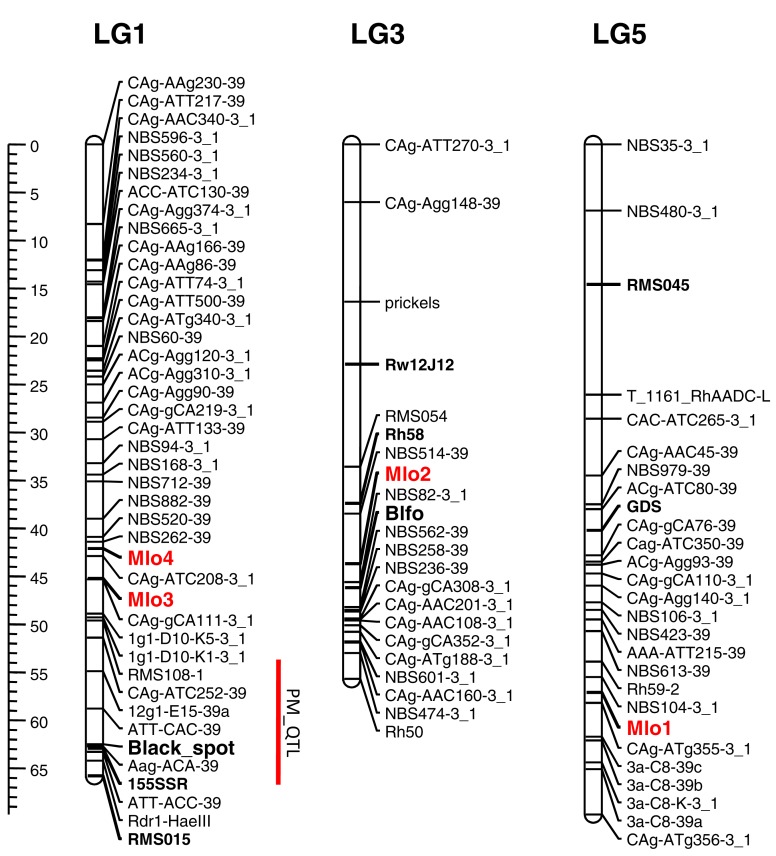
**Genetic linkage map of the linkage groups 1, 3, and 5 harboring the *MLO* genes**. The linkage groups of the parent 95/13-39 of the population 97/7 are shown. Map distances are given using cM as a ruler. The bridge markers that have been mapped in other rose populations (Spiller et al., 2011) are indicated in bold. The rose *MLO* genes are indicated in red. A powdery mildew QTL mapped in Linde et al., 2006 is indicated by a red bar right to linkage group 1. The plots were generated using MapChart 2.1 (Voorrips, 2002).

Knowledge of the map positions of RhMLO1–4 allows for analysis concerning the co-localization of these genes with known powdery mildew resistance loci and the analysis of syntenic relationships with other rosaceous genomes. We mapped all four genes in the diploid segregating population 97/7, which was previously used to map powdery mildew resistance QTLs (Linde et al., 2006) and to build a consensus linkage map for rose (Spiller et al., 2011). RhMLO1 is located on LG5 between markers NBS104-3_1 and CAg-ATg355-3_1 (Figure 4). Thus far, neither resistance genes nor any other phenotypic traits have been mapped to this region. RhMLO2 is located on LG3 near the double flower locus *Blfo*. RhMLO3 and RhMLO4 are closely linked to one another on LG1 near the *Rdr1* black spot resistance gene. On the consensus map, genes for citronellol and geraniol biosynthesis have been mapped to the same region. A QTL for powdery mildew resistance was mapped to this region by Linde et al. (2006). This weak QTL was specific for only 2 years of evaluation under greenhouse conditions.

### Comparison of the Rh*MLO* sequences to *F. vesca* and *P. persica* *MLO* sequences

Complete genome sequences are available for several species of the *Rosaceae*, including *F. vesca*, *Malus domestica*, and *P. persica* (Sosinski et al., 2010; Velasco et al., 2010; Shulaev et al., 2011). We conducted BLAST analyses of the four RhMLO sequences against the predicted proteins and the genomic sequences of the closest rose relative *F. vesca* as well as *P. persica* and we identified homologous genes in both plants (Table 2). One sequence corresponding to RhMLO1 was found in strawberry and peach respectively. The same situation was found for RhMLO2. Both genotypes possess only one homolog to both RhMLO3 and 4. RhMLO1 corresponds to ppa003207m on *Prunus* linkage group 6 and to gene02774 of *Fragaria*, which is not yet assigned to a pseudo molecule in the genome assembly v1.0. The *Prunus* linkage group 6 is partly syntenic to the linkage group 3 of the *Fragaria* genome. The *Fragaria* linkage group 3 is syntenic to the rose linkage group 5 (Gar et al., 2011), which corresponds to the location we obtained in our mapping experiment. RhMLO2 is homologous to ppa003437m on the *Prunus* linkage group 6 in a region syntenic to a position on linkage group 6 of *Fragaria*. The *Fragaria* homolog of RhMLO2, gene09653, is located on pseudo molecule 6, as expected. The RhMLO3/4 homologs are the peach sequence ppa003466m on linkage group 2 and the strawberry sequence gene23198 on pseudo molecule 7. These locations are also in accordance with known syntenic correlations (Vilanova et al., 2008). As neither peach nor the close relative strawberry exhibit two related and linked genes at this position, a recent gene duplication event can be assumed.

**Table 2:** **Homologs of RhMLO sequences in the *Fragaria vesca* and *Prunus persica* genomes**.

Rose MLO	*Fragaria vesca*			*Prunus persica*		
	Gene prediction (hybrid transcript)	NCBI BLASTn *E* value	Scaffold/Pseudo molecule in genome v1.0	Gene prediction	NCBI BLASTn *E* value	Pseudo molecule in peach v1.0
RhMLO1	mRNA02774	0.0	Scf0512887/−	Ppa003207m	0.0	Scf6
RhMLO2	mRNA09653	0.0	Scf0513149/LG6	Ppa003437m	0.0	Scf6
RhMLO3	mRNA23198	0.0	Scf0513008/LG7	Ppa003466m	−179	Scf2
RhMLO4	mRNA23198	0.0	Scf0513008/LG7	Ppa003466m	−153	Scf2

We also used the strawberry and peach genome sequences to test whether we had isolated all *MLO* orthologs expressed in rose. Therefore, we performed BLASTx searches against the genomes of *Prunus* and *Fragaria* using AtMlo2, 6, and 12, SlMlo1 and PsMlo1 as queries. All analyses identified the same set of three sequences matching RhMLO1, 2, 3, and 4. We conclude that we have likely identified all of the *MLO* orthologs of *Rosa* involved in the rose powdery mildew interaction.

## Discussion

*Mildew resistance locus o* genes have been identified in a number of species based on their sequence similarity to the well-characterized genes from barley and *Arabidopsis* (Yu et al., 2005; Feechan et al., 2008; Liu and Zhu, 2008; Cheng et al., 2012). Only a subset of these sequences have been functionally characterized and shown to be involved in plant-pathogen interactions. In addition to the first *MLO* gene isolated from barley (Buschges et al., 1997), orthologs from *Arabidopsis*, tomato, and pea have been described (Consonni et al., 2006; Bai et al., 2008; Humphry et al., 2011; Pavan et al., 2011).

Here, we present the isolation and the first characterization of four *MLO* genes from roses. These genes have homology to the *MLO* genes involved in the interactions of plants with powdery mildews. All *MLO* genes from dicots for which a function in this interaction has been experimentally demonstrated fall into one phylogenetic clade. This clade consists of the *Arabidopsis* genes AtMLO2, 6, and 12, the tomato gene SlMLO1 and the pea gene PsMLO1. With most phylogenetic methods, the barley gene HvMLO1 builds a closely related cluster (Feechan et al., 2008). All four rose genes fall into the dicot cluster for powdery mildew related MLOs and are therefore candidate *MLO* orthologs. Their position within this cluster is highly supported by bootstrap values and is reproducible with a range of computational methods, including maximum likelihood and minimum evolution trees (data not shown). The presence of sequence motifs known to be indispensable for MLO function also supports a role of these genes in mildew susceptibility. The calmodulin-binding site is conserved in all four sequences. Another motif at the C-terminus, the consensus sequence D/E-F-S/T-F, is found in all MLOs. This motif is conserved in RhMLO1 and 2 but varies in RhMLO3 and RhMLO4 by one amino acid (S-F-S-F instead of D/E-F-S/T-F). Whether this variation excludes RhMLO3 and RhMLO4 from being functional in the rose powdery mildew interaction is not yet clear. Due to the small number of functional analyses among MLO homologs, these genes may simply be another variation on the common scheme.

*MLO* genes are characterized by a large number of exons, ranging from 12 to 15. All four rose *MLO* genes contain 15 exons and fall within the structural range of the other *MLO* genes. Very large introns in MLO1 and in MLO3, due to a retroposon insertion, are correctly spliced but have not been reported in other species to date. In pea, a large insertion is assumed to cause a loss-of-function in the line JI2302 (Humphry et al., 2011). The extent of sequence variability in *MLO* genes must be considered when a screening strategy for the identification of mutations is selected. A classical TILLING approach can be pursued with highly uniform sequences; therefore, the sequence comparison of rose *MLO* alleles was performed for three diploid *R. multiflora* hybrids and one tetraploid rose variety. The number of alleles and their sequence similarities differ widely between the four MLO genes. MLO2, with only two alleles and six SNPs, appears to be the most conserved *MLO*. MLO1, 3, and 4 possess both more alleles and more SNPs within these alleles. Whether or not this difference in conservation is due to selective constraints is difficult to judge due to the small sample size. One possible explanation is that the gene for recurrent flowering in roses is located on the same linkage group as MLO2. As this trait is speculated as being derived from only a few genetic sources, this phenomenon may explain the lack of genetic diversity for MLO2. Even with the limited amount of data, the level of polymorphism is too high for a conventional TILLING approach. Next-generation sequencing will be the method of choice for detecting putative loss-of-function mutations in each rose candidate gene.

The rose *MLO* genes map to three different linkage groups on the rose chromosome map. RhMLO1 and RhMLO2 do not co-localize with any genes involved in plant-pathogen interactions that have been identified thus far. The position of RhMLO2 is close to the dominant gene for double flowers, which has two consequences. First, commercial cut and pot rose breeding is exclusively aimed at double flowered varieties. Most of the garden rose varieties are also double flowered. To select particular alleles from this *MLO* locus, recessive alleles of the double flower locus have to be in linkage configuration. Recombinants between the two loci would have to otherwise be selected to combine the correct *MLO* allele with the dominant allele at the double flower locus. Second, a gene for self-incompatibility is located on the same chromosome arm. This phenomenon has consequences for the selection of *MLO* and double flowers in diploid progeny, as it leads to distorted segregation. RhMLO3 and RhMLO4 are closely linked at the same position on linkage group 1. Despite the fact that both genes share a high degree of sequence similarity at the DNA level, recombination between them confirms the presence of two separate genes. This is in agreement with the observed number of alleles in two diploid rose genotypes. Published data on the resistance to powdery mildew places a weak QTL close to the position of RhMLO3 and 4 (Linde et al., 2006). A functional link between these observations lacks sufficient evidence, as these data have been generated with partially overlapping data sets, and the QTL positions are not as precisely determined as single major genes.

Knowledge concerning the map positions of the rose *MLO* genes will be important for *MLO*-based resistance breeding in two ways. First, it allows the selection of mutated *MLO* alleles in segregating progeny and facilitates the determination of copy number for *MLO* alleles. Second, gene pyramiding can be facilitated with known map positions in the case that more than one gene must be mutated to obtain mildew resistance.

Powdery mildew is one of the most important diseases of roses worldwide. Legal restrictions for the use of fungicides and consumer concerns limit the application of fungicides in large-scale production greenhouses. Previous results indicate that a large number of pathovars exist that, in combination with sexual recombination and a high mobility by airborne conidia, will lead to the quick adaptation of pathogen populations to single-dominant resistance genes. Thus, single-dominant resistance genes will be of limited use in developing resistant rose cultivars. The use of QTLs for resistance poses other problems. The tetraploid nature of most varieties leads to very complex patterns of inheritance and difficulties in the marker-assisted selection of resistance QTLs. A single mutated locus that, if homozygous, confers broad-spectrum resistance would be an interesting alternative. Although it will be difficult to select homozygous recessive progeny in tetraploid populations, the example of the recurrent flowering locus that has been utilized in rose breeding for more than a century demonstrates that this method can be successful. A homozygous recessive mutation in a *TFL* ortholog (Iwata et al., 2012) changes the seasonal flowering habit of rose cultivars into continuous flowering over the entire growing period.

The functional characterization of *MLO* genes involved in plant powdery mildew interactions has been performed by the complementation of natural or induced *MLO* mutants. In rose, no *MLO* mutants are currently known. One possible solution to this problem is a heterologous expression system in a species with characterized *MLO* genes for which mutants are available. Although the complementation of *MLO* mutants with wild type genes across classes of flowering plants (dicots vs. monocots) has not been successful, a number of cases where complementation is possible have been reported within each class (Elliott et al., 2002). Pea mutants can be complemented with *MLO* genes from *Medicago* (Humphry et al., 2011), though with a reduced efficiency that is apparent in a lower rate of haustoria formation as compared to complementation with genes from the same species. Characterization of the rose *MLO* homologs by heterologous complementation in *Arabidopsis* is currently underway in cooperation with other groups.

Also, we are currently using a reverse genetics approach to identify naturally occurring loss-of-function mutants within a large collection of rose genotypes. Such plants will be used in crosses to generate homozygous recessive loss-of-function mutants for individual *MLO* genes that can then be studied for their response to rose powdery mildew.

### GenBank accession numbers

The sequences of *RhMLO1–RhMLO4* were deposited under GenBank accession numbers JX847131–JX847134.

## Conflict of Interest Statement

The authors declare that the research was conducted in the absence of any commercial or financial relationships that could be construed as a potential conflict of interest.

## Footnotes

^1^http://frodo.wi.mit.edu/primer3/

^2^ http://blast.ncbi.nlm.nih.gov

^3^ http://www.rosaceae.org

^4^ http://www.ebi.ac.uk/clustalw

## Appendix

**Table A1:** **Primers used in the amplification of rose *MLO* sequences**.

MLO	Primer	Sequence	Application	Annealing temperature (°C)
RhMLO1	57F2	tccctcagtccagctttctctc	Full length amplification	59
	57R2	atcgccgtcgtggttgtaat		
	MLO1P3F	gccctcgcctcattctctac	Mapping	59
	MLO1P3R	ccatgcaaagaacgcaagt	
RhMLO2	26981F2	gcactaaccaaaacacaaacacc	Full length amplification	58
	34824R2	tctcccattgcaaagcttatt		
	MLO2kartF3	gcagccggatatgacaaatg	Mapping	58
	MLO2kartR3	tgaagctggtgaatcccata	
RhMLO3	C650_55F	tccaatggaacttgaatatgagc	cDNA amplification	60
	12306R1	gccatgtttctgcaaatgct		
	C650_55F	tccaatggaacttgaatatgagc	Genomic 5′part	59
	12309B	agtgtgtaaacctcaccccatt		
	MLO3gen_F1	cttccaccaaacccctgaag	Genomic 3′part	61
	MLO3_stopR	caactctttcactttcttgatg		
	Mlo_micF1	gggattcaccagctccatgt	Mapping	63
	Mlo_micR	tgtctcattctcccaatacttcca	
RhMLO3	5RACEC650_1	gccggaaactgaagacagaat	5′RACE	60
RhMLO4	5RACEC650_2	gacaggaatcccaacagcat		
	3RACEC650_1	aaccatgatggggatgagga	3′RACE	62
	3RACEC650_2	ggggatgaggatccaggaaa	
RhMLO4	5′21b	ggttttcttgtgagttcttttgag	Full length amplification	60
	12305R2	aacaccaagcctaaaggagca		
	Mlo4F2207	ctatggccaccttatatcag	Mapping	60
	Mlo4R2607	aattaagatccctctgattgg		

**Table A2:** **Pairwise amino acid identities between the rose *MLO* genes and *M LO* genes from other species used in the phylogenetic analysis**.

Seq→	Rh MLO1	Rh MLO2	Rh MLO3	Rh MLO4	Hv MLO	Sl MLO1	Ps MLO1	At MLO2	At MLO6	At MLO12	At MLO1	At MLO3	At MLO4	At MLO5	At MLO7	At MLO8	At MLO9	At MLO10	At MLO11	At MLO13	At MLO14	At MLO15
RhMLO1	ID																					
RhMLO2	0,64	ID																				
RhMLO3	0,55	0,55	ID																			
RhMLO4	0,53	0,54	0,92	ID																		
HvMLO	0,41	0,40	0,42	0,41	ID																	
SlMLO1	0,52	0,51	0,53	0,52	0,42	ID																
PsMLO1	0,53	0,54	0,55	0,53	0,40	0,50	ID															
AtMLO2	0,55	0,53	0,60	0,59	0,42	0,51	0,54	ID														
AtMLO6	0,55	0,54	0,59	0,58	0,42	0,50	0,53	0,75	ID													
AtMLO12	0,55	0,55	0,60	0,58	0,42	0,52	0,57	0,67	0,65	ID												
AtMLO1	0,33	0,32	0,33	0,33	0,34	0,33	0,33	0,32	0,32	0,32	ID											
AtMLO3	0,35	0,34	0,36	0,35	0,35	0,36	0,35	0,34	0,34	0,36	0,34	ID										
AtMLO4	0,27	0,25	0,26	0,26	0,25	0,25	0,27	0,26	0,27	0,27	0,30	0,26	ID									
AtMLO5	0,39	0,37	0,37	0,37	0,39	0,38	0,38	0,37	0,37	0,36	0,35	0,36	0,26	ID								
AtMLO7	0,37	0,36	0,36	0,35	0,38	0,35	0,35	0,36	0,36	0,34	0,33	0,36	0,26	0,48	ID							
AtMLO8	0,37	0,36	0,38	0,38	0,37	0,33	0,36	0,35	0,37	0,35	0,30	0,32	0,25	0,45	0,53	ID						
AtMLO9	0,40	0,37	0,38	0,37	0,38	0,37	0,37	0,36	0,37	0,36	0,36	0,36	0,25	0,78	0,47	0,44	ID					
AtMLO10	0,39	0,38	0,37	0,37	0,39	0,35	0,37	0,36	0,39	0,36	0,31	0,35	0,26	0,46	0,67	0,58	0,44	ID				
AtMLO11	0,29	0,28	0,28	0,28	0,27	0,27	0,29	0,26	0,28	0,26	0,31	0,27	0,40	0,30	0,29	0,29	0,30	0,29	ID			
AtMLO13	0,34	0,32	0,32	0,32	0,32	0,32	0,33	0,33	0,32	0,33	0,47	0,32	0,29	0,35	0,31	0,30	0,37	0,29	0,30	ID		
AtMLO14	0,30	0,29	0,28	0,28	0,28	0,28	0,30	0,28	0,29	0,27	0,33	0,27	0,39	0,30	0,29	0,29	0,30	0,29	0,78	0,32	ID	
AtMLO15	0,34	0,33	0,34	0,33	0,33	0,33	0,33	0,33	0,33	0,32	0,55	0,31	0,27	0,36	0,32	0,31	0,37	0,31	0,30	0,49	0,33	ID

## References

[B1] Altschul S. F. Madden T. L. Schaffer A. A. Zhang J. H. Zhang Z. Miller W. (1997). Gapped BLAST and PSI-BLAST: a new generation of protein database search programs. Nucleic Acids Res. 25, 3389–3402 10.1093/nar/25.17.3389 9254694 PMC146917

[B2] Bai Y. Pavan S. Zheng Z. Zappel N. F. Reinstaedler A. Lotti C. (2008). Naturally occurring broad-spectrum powdery mildew resistance in a central American tomato accession is caused by loss of Mlo function. Mol. Plant Microbe Interact. 21, 30–39 10.1094/MPMI-21-1-0030 18052880

[B3] Bhat R. A. Miklis M. Schmelzer E. Schulze-Lefert P. Panstruga R. (2005). Recruitment and interaction dynamics of plant penetration resistance components in a plasma membrane microdomain. Proc. Natl. Acad. Sci. U.S.A. 102, 3135–3140 10.1073/pnas.0500012102 15703292 PMC549507

[B4] Biber A. Kaufmann H. Linde M. Spiller M. Terefe D. Debener T. (2010). Molecular markers from a BAC contig spanning the Rdr1 locus: a tool for marker-assisted selection in roses. Theor. Appl. Genet. 120, 765–773 10.1007/s00122-009-1197-9 19911159

[B5] Buschges R. Hollricher K. Panstruga R. Simons G. Wolter M. Frijters A. (1997). The barley mlo gene: a novel control element of plant pathogen resistance. Cell 88, 695–705 10.1016/S0092-8674(00)81912-1 9054509

[B6] Cheng H. Kun W. Liu D. Su Y. He Q. (2012). Molecular cloning and expression analysis of CmMlo1 in melon. Mol. Biol. Rep. 39, 1903–1907 10.1007/s11033-012-1824-4 21660472

[B7] Collins N. C. Thordal-Christensen H. Lipka V. Bau S. Kombrink E. Qiu J. L. (2003). SNARE-protein-mediated disease resistance at the plant cell wall. Nature 425, 973–977 10.1038/nature02076 14586469

[B8] Consonni C. Humphry M. E. Hartmann H. A. Livaja M. Durner J. Westphal L. (2006). Conserved requirement for a plant host cell protein in powdery mildew pathogenesis. Nat. Rev. Genet. 38, 716–720 10.1038/ng1806 16732289

[B9] Debener T. Linde M. (2009). Exploring complex ornamental genomes: the rose as a model plant. CRC Crit. Rev. Plant Sci. 28, 267–280 10.1080/07352680903035481

[B10] Devoto A. Hartmann H. A. Piffanelli P. Elliott C. Simmons C. Taramino G. (2003). Molecular phylogeny and evolution of the plant-specific seven-transmembrane MLO family. J. Mol. Evol. 56, 77–88 10.1007/s00239-002-2382-5 12569425

[B11] Devoto A. Piffanelli P. Nilsson I. Wallin E. Panstruga R. Heijne G. (1999). Topology, subcellular localization, and sequence diversity of the Mlo family in plants. J. Biol. Chem. 274, 34993–35004 10.1074/jbc.274.49.34993 10574976

[B12] Dugo M. L. Satovic Z. Millán T. Cubero J. I. Rubiales D. Cabrera A. (2005). Genetic mapping of QTLs controlling horticultural traits in diploid roses. Theor. Appl. Genet. 111, 511–520 10.1007/s00122-005-2042-4 15905992

[B13] Elliott C. Zhou F. S. Spielmeyer W. Panstruga R. Schulze-Lefert P. (2002). Functional conservation of wheat and rice Mlo orthologs in defense modulation to the powdery mildew fungus. Mol. Plant Microbe Interact. 15, 1069–1077 10.1094/MPMI.2002.15.10.1069 12437305

[B14] Feechan A. Jermakow A. M. Torregrosa L. Panstruga R. Dry I. B. (2008). Identification of grapevine MLO gene candidates involved in susceptibility to powdery mildew. Funct. Plant Biol. 35, 1255–1266 10.1071/FP08173 32688872

[B15] Gar O. Sargent D. J. Tsai C.-J. Pleban T. Shalev G. Byrne D. H. (2011). An autotetraploid linkage map of rose (*Rosa hybrida*) validated using the strawberry (*Fragaria* *vesca*) genome sequence. PLoS ONE 6, e20463 10.1371/journal.pone.0020463 21647382 PMC3103584

[B16] Hall T. (1999). BioEdit: a user-friendly biological sequence alignment editor and analysis program for Windows 95/98/NT. Nucleic Acids Symp Ser (Oxf.) 41, 95–98

[B17] Hosseini Moghaddam H. Leus L. Riek J. de van Huylenbroeck J. van Bockstaele E. (2012). Construction of a genetic linkage map with SSR, AFLP, and morphological markers to locate QTLs controlling pathotype-specific powdery mildew resistance in diploid roses. Euphytica 184, 413–427 10.1007/s10681-011-0616-6

[B18] Humphry M. Consonni C. Panstruga R. (2006). mlo-Based powdery mildew immunity: silver bullet or simply non-host resistance? Mol. Plant Pathol. 7, 605–610 10.1111/j.1364-3703.2006.00362.x 20507473

[B19] Humphry M. Reinstaedler A. Ivanov S. Bisseling T. Panstruga R. (2011). Durable broad-spectrum powdery mildew resistance in pea er1 plants is conferred by natural loss-of-function mutations in PsMLO1. Mol. Plant Pathol. 12, 866–878 10.1111/j.1364-3703.2011.00718.x 21726385 PMC6640514

[B20] Iwata H. Gaston A. Remay A. Thouroude T. Jeauffre J. Kawamura K. (2012). The TFL1 homologue KSN is a regulator of continuous flowering in rose and strawberry. Plant J. 69, 116–125 10.1111/j.1365-313X.2011.04776.x 21895811

[B21] Jorgensen J. H. (1992). Discovery, characterization and exploitation of MLO powdery mildew resistance in barley. Euphytica 63, 141–152 10.1007/BF00023919

[B22] Kim M. C. Lee S. H. Kim J. K. Chun H. J. Choi M. S. Chung W. S. (2002). Mlo, a modulator of plant defense and cell death, is a novel calmodulin-binding protein – Isolation and characterization of a rice Mlo homologue. J. Biol. Chem. 277, 19304–19314 10.1074/jbc.M200835200 11904292

[B23] Kurowska M. Daszkowska-Golec A. Gruszka D. Marzec M. Szurman M. Szarejko I. (2011). TILLING – a shortcut in functional genomics. J. Appl. Genet. 52, 371–390 10.1007/s13353-011-0061-1 21912935 PMC3189332

[B24] Leus L. Dewitte A. van Huylenbroeck J. Vanhoutte N. van Bockstaele E. Höfte M. (2006). *Podosphaera pannosa* (syn. *Sphaerotheca* *pannosa*) on *Rosa* and *Prunus* spp.: characterization of pathotypes by differential plant reactions and ITS sequences. J. Phytopathol. 154, 23–28

[B25] Linde M. Debener T. (2003). Isolation and identification of eight races of powdery mildew of roses (*Podosphaera pannosa*; Wallr.: Fr.) de Bary and the genetic analysis of the resistance gene Rpp1. Theor. Appl. Genet. 107, 256–262 10.1007/s00122-003-1240-1 12845441

[B26] Linde M. Hattendorf A. Kaufmann H. Debener T. (2006). Powdery mildew resistance in roses: QTL mapping in different environments using selective genotyping. Theor. Appl. Genet. 113, 1081–1092 10.1007/s00122-006-0367-2 16896710

[B27] Linde M. Mattiesch L. Debener T. (2004). Rpp1, a dominant gene providing race-specific resistance to rose powdery mildew (*Podosphaera pannosa*): molecular mapping, SCAR development and confirmation of disease resistance data. Theor. Appl. Genet. 109, 1261–1266 10.1007/s00122-004-1735-4 15490103

[B28] Linde M. Shishkoff N. (2003). “Fungi; powdery mildew,” in Encyclopedia of Rose Science, eds Roberts A. V. Debener T. Gudin S. (Oxford: Elsevier), 158–165

[B29] Liu Q. Zhu H. (2008). Molecular evolution of the MLO gene family in *Oryza* *sativa* and their functional divergence. Gene 409, 1–10 10.1016/j.gene.2007.10.031 18155857

[B30] Marroni F. Pinosio S. Di Centa E. Jurman I. Boerjan W. Felice N. (2011). Large-scale detection of rare variants via pooled multiplexed next-generation sequencing: towards next-generation ecotilling. Plant J. 67, 736–745 10.1111/j.1365-313X.2011.04627.x 21554453

[B31] Muller K. McDermott J. M. Wolfe M. S. Limpert E. (1996). Analysis of diversity in populations of plant pathogens: the barley powdery mildew pathogen across Europe. Eur. J. Plant Pathol. 102, 385–395 10.1007/BF01878133

[B32] Orita M. Iwahana H. Kanazawa H. Hayashi K. Sekiya T. (1989). Detection of polymorphisms of human DNA by gel electrophoresis as single-strand conformation polymorphisms. Proc. Natl. Acad. Sci. U.S.A. 86, 2766–2770 10.1073/pnas.86.8.2766 2565038 PMC286999

[B33] Panstruga R. (2005). Discovery of novel conserved peptide domains by ortholog comparison within plant multi-protein families. Plant Mol. Biol. 59, 485–500 10.1007/s11103-005-0353-0 16235112

[B34] Pavan S. Schiavulli A. Appiano M. Marcotrigiano A. R. Cillo F. Visser R. G. F. (2011). Pea powdery mildew er1 resistance is associated to loss-of-function mutations at a MLO homologous locus. Theor. Appl. Genet. 123, 1425–1431 10.1007/s00122-011-1677-6 21850477

[B35] Reinstaedler A. Mueller J. Czembor J. H. Piffanelli P. Panstruga R. (2010). Novel induced mlo mutant alleles in combination with site-directed mutagenesis reveal functionally important domains in the heptahelical barley Mlo protein. BMC Plant Biol. 10, 31 10.1007/s00122-011-1677-1676 20170486 PMC2844067

[B36] Shulaev V. Sargent D. J. Crowhurst R. N. Mockler T. C. Folkerts O. Delcher A. L. (2011). The genome of woodland strawberry (*Fragaria* *vesca*). Nat. Genet. 43, 109–116 10.1038/ng.740 21186353 PMC3326587

[B37] Sosinski B. Jung S. Verde I. Schmutz J. Scholl E. Staton M. (2010). “The peach genome sequence and its utility for comparative genomics,” Proceedings of the International Conference on Plant and Animal genomes XVIII, San Diego, CA.

[B38] Spiller M. Linde M. Hibrand-Saint Oyant L. Tsai C.-J. Byrne D. H. Smulders M. J. M. (2011). Towards a unified genetic map for diploid roses. Theor. Appl. Genet. 122, 489–500 10.1007/s00122-010-1463-x 20936462

[B39] Tamura K. Peterson D. Peterson N. Stecher G. Nei M. Kumar S. (2011). MEGA5: molecular evolutionary genetics analysis using maximum likelihood, evolutionary distance, and maximum parsimony methods. Mol. Biol. Evol. 28, 2731–2739 10.1093/molbev/msr121 21546353 PMC3203626

[B40] Terefe-Ayana D. Yasmin A. Le T. L. Kaufmann H. Biber A. Kühr A. (2011). Mining disease-resistance genes in roses: functional and molecular characterization of the rdr1 locus. Front. Plant Sci. 2:35 10.3389/fpls.2011.00035 22639591 PMC3355636

[B41] Thompson J. Burdon J. (1992). Gene-for-gene coevolution between plants and parasites. Nature 360, 121–125 10.1038/360121a0

[B42] Thompson J. D. Higgins D. G. Gibson T. J. (1994). CLUSTAL W: improving the sensitivity of progressive multiple sequence alignment through sequence weighting, position-specific gap penalties and weight matrix choice. Nucleic Acids Res. 22, 4673–4680 10.1093/nar/22.22.4673 7984417 PMC308517

[B43] Tsai H. Howell T. Nitcher R. Missirian V. Watson B. Ngo K. J. (2011). Discovery of rare mutations in populations: TILLING by sequencing. Plant Physiol. 156, 1257–1268 10.1104/pp.110.169748 21531898 PMC3135940

[B44] van Ooijen J. W. (2006). JoinMap 4 Manual: Software for the Calculation of Genetic Linkage Maps in Experimental Populations. Wageningen: Kyazma B. V.

[B45] Velasco R. Zharkikh A. Affourtit J. Dhingra A. Cestaro A. Kalyanaraman A. (2010). The genome of the domesticated apple (Malus × domestica Borkh.) Nat. Genet. 42, 833–839 10.1038/ng.654 20802477

[B46] Vilanova S. Sargent D. J. Arus P. Monfort A. (2008). Synteny conservation between two distantly-related Rosaceae genomes: Prunus (the stone fruits) and Fragaria (the strawberry). BMC Plant Biol. 8, 67 10.1186/1471-22229-8-67 18564412 PMC2442709

[B47] Voorrips R. E. (2002). MapChart: software for the graphical presentation of linkage maps and QTLs. J. Hered. 93, 77–78 10.1093/jhered/93.1.77 12011185

[B48] Xu Q. Wen X. P. Deng X. X. (2005). Isolation of TIR and nonTIR NBS-LRR resistance gene analogues and identification of molecular markers linked to a powdery mildew resistance locus in chestnut rose (*Rosa roxburghii Tratt*). Theor. Appl. Genet. 111, 819–830 10.1007/s00122-005-0027-y 16075209

[B49] Yan Z. Denneboom C. Hattendorf A. Dolstra O. Debener T. Stam P. (2005). Construction of an integrated map of rose with AFLP, SSR, PK, RGA, RFLP, SCAR, and morphological markers. Theor. Appl. Genet. 110, 766–777 10.1007/s00122-004-1903-6 15672277

[B50] Yu L. Niu J. S. Chen P. D. Ma Z. Q. Liu D. J. (2005). Cloning, physical mapping and expression analysis of a wheat mlo-like. J. Integr. Plant Biol. 47, 214–222 10.1111/j.1744-7909.2005.00030.x

